# Items

**Published:** 1898-09

**Authors:** 


### ITEMS.

Commenting upon the fact that Prof. Behring has succeeded recently in obtaining in this country a patent upon diphtheria antitoxin and that all firms that have been engaged in the manufacture of this highly useful therapeutic agent have been served with a notice to discontinue their work in that direction, the *Maryland Medical Journal* very justly remarks as follows: "Physicians, unlike business men, will calmly submit to almost any indignity, because they are too apathetic to act, but it is a pleasure to see that the reputable firms in this country which have invested large sums in valuable plants for making this serum have agreed to fight the Behring patent, and it is the duty of the profession of the United States to throw their weight with these firms and to use their products as long as they are found to be worth using."

---

A recent order issued from the headquarters of the First Division, Third Corps, at Chickamauga Park, is of more than usual interest because it shows the efforts being made in at least one command to give soldiers thorough instruction in hygiene. Sometime ago, says the *Buffalo Express*, the surgeon-general gave Lieut.-Col. Charles Smart full authority to endeavor to carry out the reform. Col. Smart prepared a manual, which is entitled, How to keep well. The general order referred to directs that, for the purpose of instruction in this manual, company commanders divide their companies into squads, with a noncommissioned officer in charge of each and that this officer be required to assemble his men at regular intervals, instruct them in the manual and afterwards quiz the soldiers on the information given. This process is to be continued until every man in the division understands the rules laid down by Col. Smart. In connection with this plan for instruction a special diet kitchen has been established at the First Division Hospital. When more of these kitchens have been put in operation it is expected that from them general supervision will be exercised over the company kitchens. This dietary movement is the work of a woman, Mrs. John L. Hogan, who certainly deserves praise for the practical character of her plans. Whatever contributions may be made to this service should be in the form of money and should be sent to the surgeon-general.

---

THE FIRST NATHAN LEWIS HATFIELD PRIZE FOR ORIGINAL RESEARCH IN MEDICINE. —The College of Physicians of Philadelphia announces through its committee that the sum of $500 will be awarded to the author of the best essay in competition for the above prize.

Subject: A pathological and clinical study of the Thymus gland and its relation. Essays must be submitted on or before January 1, 1900. Each essay must be typewritten, designated by a motto or device and accompanied by a sealed envelope bearing the same motto or device and containing the name and address of the author. No envelope will be opened except that which accompanies the successful essay.

The committee will return the unsuccessful essays if reclaimed by their respective writers or their agents within one year, and reserves the right not to make an award if no essay submitted is considered worthy of the prize.

The treatment of the subject must, in accordance with the conditions of the Trust, embody original observations, or researches or original deductions. The competition shall be open to members of the medical profession and men of science in the United States. The original of the successful essay shall become the property of the College of Physicians. The trustees shall have full control of the publication of the memorial essay. It shall be published in the transactions of the college and also when expedient as a separate issue. Address J. C. Wilson, M. D., Chairman, College of Physicians, 219 South Thirteenth street, Philadelphia, Pa.

THE PURIFICATION OF SANTIAGO.—Whatever may truthfully be placed to the discredit of Gen. Ben Butler during his military rule of New Orleans (*Cleveland Medical Gazette*), it will always be remembered to his credit that he found the city dirty and left it clean; he found it the chosen home of yellow fever and he banished the pestilence. He showed the way and successive municipal administrations have walked in it ever since, to the great gain of the city in health, reputation and prosperity. What was done in New Orleans under the rigor of military administration can be done in Santiago through similar authority and probably with less harshness. Santiago is unspeakably filthy, not simply as the result of war conditions, but from generations, centuries of neglect of the most ordinary sanitary precautions.—*Cleveland Plain Dealer.*

THE DIFFICULTIES OF THE BRITISH ARMY MEDICAL OFFICER.—A correspondent signing himself P. O., writing to the *Indian Lancet* for July 1st on the Future of the army medical staff, says: The concession of military titles is an item of their claim; but they have other needs than this. Lord Landsdowne cannot really think that he has done all that is necessary when he abolishes the designation which connects medical men with the profession to which they have the honor to belong. The rates of pay and allowances are none too liberal—a veterinary lieutenant is more highly paid than a surgeon lieutenant—while the treatment to which medical officers are subjected in regard to changes of station is little less than scandalous. The tour of service is now running a short two years at home as against a long five years' abroad; and what is worse, during the home tour there is no fixity of tenure. A surgeon may be in Ireland one week, in Scotland the next, and perhaps in the south of England before the month is out. If he should be married, as he generally is, and takes and furnishes a house, as he often weakly does, it almost surely follows that he is ordered off somewhere else at a few days' notice. All this is bad enough for him, but it may be even worse for his patients. The other day, in a garrison town, an officer had measles in his family. The family were attended by five different medical officers during six successive days, and if the children got well it was because the captain who was their father—albeit he was a captain without being equally a medical man—doctored them himself. What medical officers most need is considerate and reasonable treatment from headquarters and considerate and reasonable treatment from those with whom they live in daily association.—*New York Medical Journal.*

